# Protein kinase B is involved in bisphenol A-induced macrophage polarization through mechanistic target of rapamycin-dependent autophagy

**DOI:** 10.1515/jtim-2026-0029

**Published:** 2026-03-06

**Authors:** Kailu Wang, Wenshuo Wei, Jingjing Wang, Yan Cong, Hanlin Wang, Chongming An, Xiaoxia Jin, Xiucong Pei

**Affiliations:** Department of Toxicology, School of Public Health, Shenyang Medical College, Shenyang, Liaoning Province, China; Community Health Service Center of Daxing New District, Xi'an, Shaanxi Province, China; Department of Occupational and Environmental Health, School of Public Health, Shenyang Medical College, Shenyang, Liaoning Province, China

## To the editor

Bisphenol A (BPA), a widespread environmental endocrine disruptor (EED), enters the human body *via* the food chain or direct contact and influences the pathogenesis of multiple diseases, including tumors.^[1]^ As the primary effector cells of the immune response, macrophages polarize into M1 or M2 phenotypes and secrete substantial quantities of pro-inflammatory or immunosuppressive cytokines in response to external stimuli, thereby regulating the microenvironment.^[2]^ Immune homeostasis is maintained only when M1/M2 polarization is in equilibrium. Exposure to BPA promotes a tumor-supportive microenvironment by disrupting the M1/M2 macrophage balance, but the mechanism is still unclear. Protein kinase B (AKT) integrates inputs from growth factors and metabolic effectors to mediate multifunctional signaling targets *via* directly phosphorylating substrates, which mediate cell proliferation, polarization and inflammation.^[3,4]^ It exists in three isoforms (AKT1, AKT2, and AKT3), which play distinct roles in tumors based on differences in their residues.^[5]^ As an important downstream molecule of AKT, mechanistic target of rapamycin (mTOR) is a key regulator of macrophage polarization, which inhibits the process of catabolism, including autophagy. Furthermore, mTOR may negatively mediate autophagy, which is a cellular self-digestion pathway that removes damaged cellular components through sequestration in double-membrane vesicles and subsequent fusion with lysosomes for acidic degradation. This study aimed to investigate the disparate roles of AKT subtypes in BPA-induced macrophage polarization and their functional interplay with mTOR-dependent autophagy in a lipopolysaccharide (LPS)-induced macrophage activation model. These findings offer novel perspectives on the impact of EEDs on the tumor microenvironment.

To investigate these mechanisms, mouse mononuclear macrophage leukemia cells (RAW264.7) were pretreated with LPS (10 μg/mL) for 30 min and then incubated with BPA (0, 10, 100, 200 μmol/L) for 12 h. Pretreatment with AKT inhibitor perifosine (PE, 5 μmol/L) or mTOR inhibitor rapamycin (RAPA, 1.37 μmol/L) for 30 min was followed by BPA (100 μmol/L) exposure. Based on the key targets identified through bioinformatics analysis, cells were also transfected with small interfering RNA targeting AKT1 (siAKT1) or AKT2 (siAKT2) (Supplementary Table S1), respectively. Cell viability, mRNA and protein levels of polarization markers, cytokines, autophagy markers, and signaling molecules were measured.

As is well known, macrophage polarization is a well-established regulator of the tumor microenvironment and represents a potential therapeutic strategy for malignancies such as acute myeloid leukemia. BPA has been reported to promote the polarization of primary Kupffer cells to pro-inflammatory M1 dominant subtypes.^[6]^ In this study, BPA significantly increased the expression of M1 markers (cluster of differentiation 86 [CD86], inducible nitric oxide synthase [iNOS]) and pro-inflammatory cytokines (tumor necrosis factor-α [TNF-α], interleukin-6 [IL-6]), while decreasing the expression of M2 markers (CD206, arginase-1 [Arg-1]) in LPS-activated macrophages (Supplementary Figure S1), which is consistent with a previous study.^[7]^ And similar effects occurred in both inactivated microglia as the resident macrophages of the brain and adipose tissue macrophages in mice fed with a high-fat diet.^[8]^ Together, these data suggested that BPA substantially altered the macrophage polarization balance in both activated and resting macrophages.

To gain more insight into the mechanism of BPA-induced macrophage polarization, we used bioinformatics to identify the joint target genes of BPA and macrophage polarization. The analysis identified 88 common targets shared by BPA and macrophage polarization (Supplementary Figure S2). Kyoto Encyclopedia of Genes and Genomes (KEGG) analysis highlighted the phosphatidylinositol 3-kinase (PI3K)/AKT and mTOR signaling pathways, inflammatory responses, and immune regulation (Supplementary Figure S3). Protein-protein interaction (PPI) network analysis of 77 interacting proteins revealed AKT1 as the most central node (Supplementary Table S2). These implied that AKT subtypes and mTOR may be involved in macrophage polarization.

Studies have shown that AKT1 and AKT2 kinase isoforms play a key role in the regulation of macrophage activation. In this study, ribonucleicacid (RNA) interference techniques were utilized for *AKT1* or *AKT2* gene knockdown to establish stably transfected cell lines in macrophages. BPA decreased AKT1 expression and increased AKT2 expression at both mRNA and protein levels (Supplementary Figure S3). Further investigation into AKT subtypes revealed that BPA enhanced the expression of CD86, iNOS, TNF-α, IL-6, and suppressed the expression of CD206, Arg-1, transforming growth factor-β (TGF-β), and IL-10 in the AKT1 knockdown cells (Supplementary Figure S4). Conversely, knockdown of AKT2 enhanced the levels of Arg-1, TGF-β, and suppressed the levels of iNOS, TNF-α (Supplementary Figure S5). Our results were not in agreement with the previous reports which might be due to the different models and activation conditions *in vivo* or *in vitro*.^[9]^ The results indicated the distinct roles for AKT1 and AKT2 in BPA-induced macrophage polarization. Moreover, AKT inhibitor PE upregulated pro-inflammatory cytokines and M1-type markers while downregulating M2-type markers, an effect consistent with that of AKT1 knockdown on BPA-induced polarization but distinct from the influence of AKT2 knockdown. Hence, our results suggested that AKT1 exerted a more critical influence than AKT2 on polarization in BPA-treated macrophages, which validated the PPI network findings.

PPE reduces AKT activity by simultaneously inhibiting phosphorylation at its Threonine 308 (Thr308) and Serine 473 (Ser473) sites. RAPA can specifically target and inhibit the mTOR signaling pathway , thereby regulating cell polarization and metabolism. Therefore, PE and RAPA were chosen to further investigate the role of autophagy in BPA-induced macrophage polarization. In this study, PE significantly enhanced BPA-induced M1 polarization and suppressed M2 polarization (Supplementary Figure S6), and inhibited phosphorylated AKT and mTOR while elevating the microtubule-associated proteins 1A/1B light chain 3 II/I (LC3II/I) ratio, indicating that AKT regulates autophagy *via* mTOR (Supplementary Figure S7). Furthermore, RAPA inhibited M1-type polarization of macrophages and promoted M2-type polarization while activating autophagy, which was similar to a previous report.^[10]^ The data suggested that mTOR-dependent autophagy regulated macrophage polarization. Collectively, the AKT/mTOR-mediated autophagy was involved in BPA-induced macrophage polarization. It should be noted that PE and RAPA exerted distinct effects on BPA-induced M1 and M2 polarization despite both enhancing autophagy, implying that additional mechanisms participate in this process.

**Figure 1:**
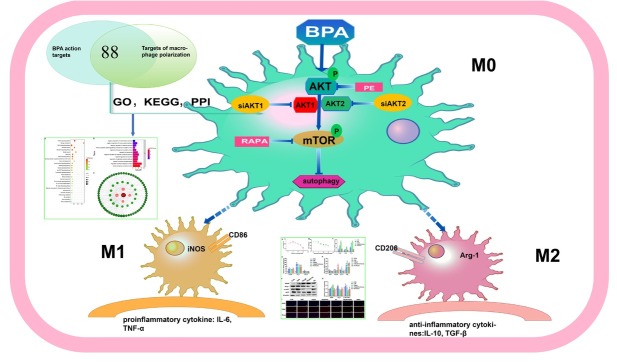
AKT mediates BPA-induced macrophage polarization through mTOR-dependent autophagy. BPA: Bisphenol A; GO: Gene Ontology; KEGG: Kyoto Encyclopedia of Genes and Genomes; PPI: protein-protein interaction; AKT: Protein kinase B; siAKT1: small interfering protein kinase B 1; mTOR: mechanistic target of rapamycin; PE: perifosine; iNOS: inducible nitric oxide synthase; CD86: cluster of differentiation 86; IL-6: interleukin-6; TNF-α: tumor necrosis factor-α; TGF-β: transforming growth factor-β.

Taken together, BPA induced the polarization of macrophages, causing an M1/M2 imbalance characterized by the upregulation of M1 markers and concurrent downregulation of M2 markers. AKT1 was found to play a major role in macrophage polarization by negatively regulating M1-type polarization while promoting M2-type polarization. The data support the hypothesis that AKT mediates BPA-induced macrophage polarization through mTOR-dependent autophagy (Figure 1). Therefore, these results provide novel insights into the specific molecular mechanisms that drive alterations in the tumor microenvironment.

## Supplementary Material

Supplementary Material Details
