# Supplementary material for: Protein kinase B is involved in bisphenol A-induced macrophage polarization through mechanistic target of rapamycin-dependent autophagy
Source: J Transl Int Med. 2026 Mar 6;14(3):485–7. doi: 10.1515/jtim-2026-0029 (PMC13320531; doi:10.1515/jtim-2026-0029)
Supplement: Supplementary file 1 — Supplementary Material Details [file jtim-2026-0029_sm.pdf]

Supplementary Table S1: The siRNA sequences used in the study.

siRNA	Orientation	Primer sequences (5′-3′)
siAKT1	Sense	GGCAGGAAGAAGAGACGAUTT
	Antisense	AUCGUCUCUUCUUCCUGCCTT
siAKT2	Sense	CCAUGAAUGACUUCGAUUATT
	Antisense	UAAUCGAAGUCAUUCAUGGTT
si-NC	Sense	UUCUCCGAACGUGUCACGUTT
	Antisense	ACGUGACACGUUCGGAGAATT

siRNA: small interfering ribonucleicacid; siAKT1: small interfering AKT1.

Supplementary Table S2: PPI ranked in the study.

Number	Gene name	Protein name	Degree
1	*AKT1*	Serine/threonine-protein kinase AKT	41
2	*ESR1*	Estrogen receptor alpha	27
3	*HSP90AA1*	Heat shock protein HSP 90-alpha	25
4	*PTGS2*	Cyclooxygenase-2	22
5	*AR*	Androgen Receptor	21
6	*BCL2L1*	Apoptosis regulator Bcl-X	17
7	*MMP9*	Matrix metalloproteinase 9	17
8	*HSP90AB1*	Heat shock protein HSP 90-beta	16
9	*ACHE*	Acetylcholinesterase	16
10	*MAP2K1*	Dual specificity mitogen-activated protein kinase kinase 1	15
11	*IGF1R*	Insulin-like growth factor I receptor	15
12	*MMP2*	Matrix metalloproteinase 2	15
13	*PRKACA*	cAMP-dependent protein kinase alpha-catalytic subunit	14
14	*DRD2*	Dopamine D2 receptor	13
15	*RAF1*	Serine/threonine-protein kinase RAF	13
16	*SLC6A4*	Serotonin transporter	13
17	*MAPT*	Microtubule-associated protein tau	13
18	*SLC6A3*	Dopamine transporter	12

19	*ADRA2A*	Alpha-2a adrenergic receptor	12
20	*DRD4*	Dopamine D4 receptor	12
21	*DRD3*	Dopamine D3 receptor	12
22	*HTR2A*	Serotonin 2a (5-HT2a) receptor	12
23	*ADRA2C*	Adrenergic receptor alpha-2	12
24	*ADRA2B*	Alpha-2b adrenergic receptor	12
25	*ALOX5*	Arachidonate 5-lipoxygenase	11
26	*HDAC1*	Histone deacetylase 1	10
27	*CYP19A1*	Cytochrome P450 19A1	10
28	*ESR2*	Estrogen receptor beta	10
29	*ADORA3*	Adenosine A3 receptor	10
30	*DRD1*	Dopamine D1 receptor	10
31	*SLC6A2*	Norepinephrine transporter	10
32	*BCL2*	Apoptosis regulator Bcl-2	10
33	*PAK1*	Serine/threonine-protein kinase PAK 1	9
34	*LTA4H*	Leukotriene A4 hydrolase	9
35	*MAPK10*	c-Jun N-terminal kinase 3	9
36	*HTR2B*	Serotonin 2b (5-HT2b) receptor	8
37	*PARP1*	Poly [ADP-ribose] polymerase-1	8
38	*CTSK*	Cathepsin K	8
39	*BRAF*	Serine/threonine-protein kinase B-raf	7
40	*HDAC6*	Histone deacetylase 6	7
41	*PTGS1*	Cyclooxygenase-1	7
42	*MMP1*	Matrix metalloproteinase 1	7
43	*ALOX15*	Arachidonate 15-lipoxygenase	7
44	*ALOX12*	Arachidonate 12-lipoxygenase	7
45	*CHRM1*	Muscarinic acetylcholine receptor M1	7
46	*HRH4*	Histamine H4 receptor	7
47	*PLA2G2A*	Phospholipase A2 group IIA	6

48	*FASN*	Fatty acid synthase	6
49	*NR1H4*	Bile acid receptor FXR	6
50	*PHLPP2*	PH domain leucine-rich repeat-containing protein phosphatase 2	5
51	*SRD5A1*	Steroid 5-alpha-reductase 1	5
52	*ADRB3*	Beta-3 adrenergic receptor	5
53	*SHBG*	Testis-specific androgen-binding protein	5
54	*DHFR*	Dihydrofolate reductase	4
55	*CHRM3*	Muscarinic acetylcholine receptor M3	4
56	*TACR2*	Neurokinin 2 receptor	4
57	*RPS6KA3*	Ribosomal protein S6 kinase alpha 3	4
58	*FGFR1*	Fibroblast growth factor receptor 1	4
59	*MYLK*	Myosin light chain kinase, smooth muscle	3
60	*HTR6*	Serotonin 6 (5-HT6) receptor	3
61	*CA2*	Carbonic anhydrase II	3
62	*TYR*	Tyrosinase	3
63	*TRAP1*	Heat shock protein 75 kDa, mitochondrial	3
64	*ROCK1*	Rho-associated protein kinase 1	2
65	*DAO*	D-amino-acid oxidase	2
66	*EPHX2*	Epoxide hydratase	2
67	*DYRK1A*	Dual-specificity tyrosine-phosphorylation regulated kinase 1A	2
68	*NR1H3*	LXR-alpha	2
69	*IDO1*	Indoleamine 2,3-dioxygenase	2
70	*CA4*	Carbonic anhydrase IV	2
71	*NR1I2*	Pregnane X receptor	2
72	*QDPR*	Dihydropteridine reductase	1
73	*DUSP3*	Dual specificity protein phosphatase 3	1
74	*CA7*	Carbonic anhydrase VII	1

75	*DYRK1B*	Dual specificity tyrosine-phosphorylation-regulated kinase 1B	1
76	*RORC*	Nuclear receptor ROR-gamma	1
77	*ALPL*	Alkaline phosphatase, tissue-nonspecific isozyme	1

PPI: protein-protein interaction.

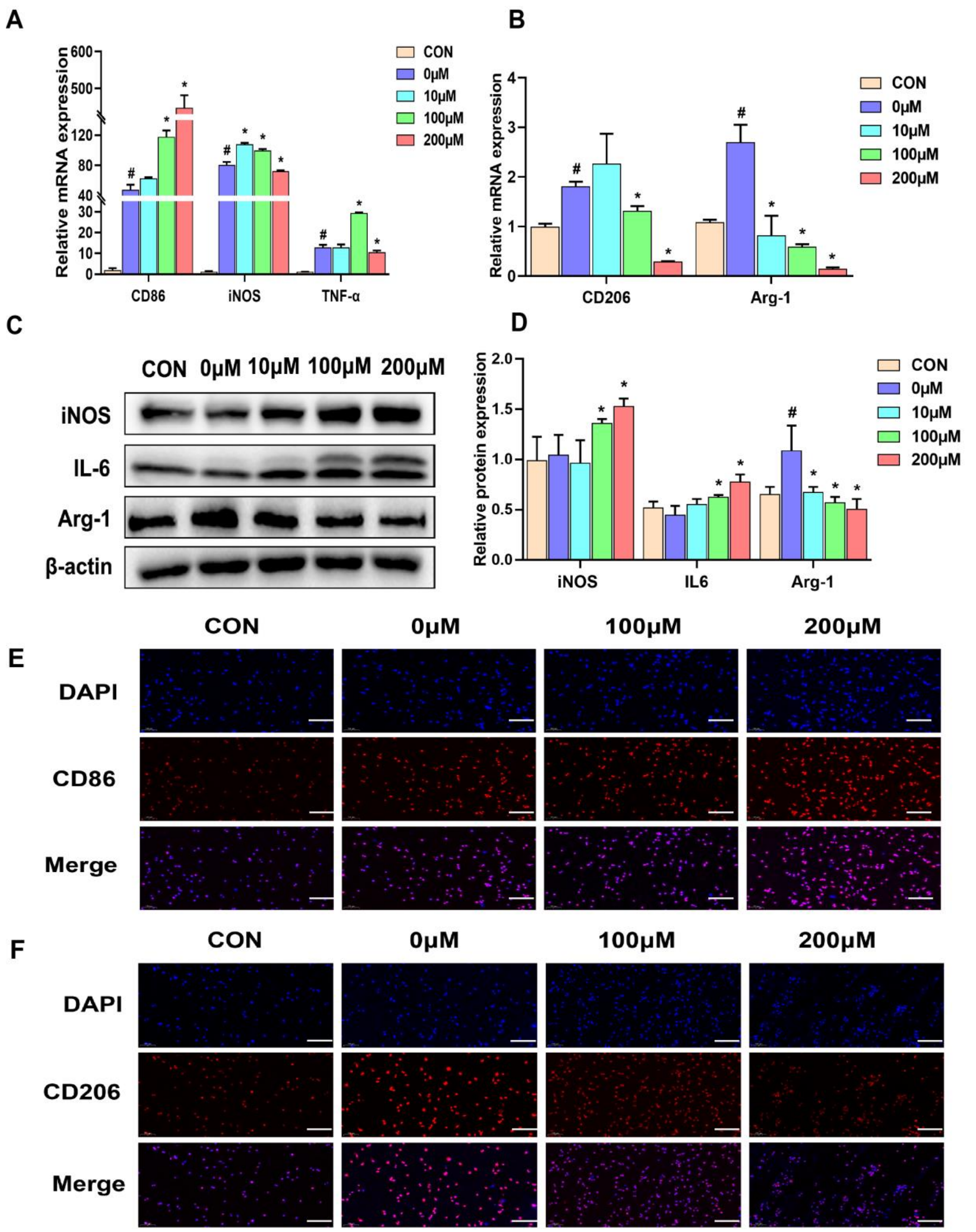

Supplementary Figure S1: Effects of BPA on polarization in macrophages. RAW264.7 cells were pretreated with 10 μg/mL LPS for 30 min followed by an additional treatment with a range of BPA concentrations (0, 10, 100 and 200 μmol/L) for 12 h. (A) The mRNA expression of M1 markers (iNOS and CD86) and pro-inflammatory factor TNF-α. (B) The mRNA expression of M2 markers (CD206 and Arg-1). (C, D) Western blot analysis of iNOS, IL-6 and Arg-1. (E, F) Immunofluorescence staining for CD86 and CD206. Scale bar represents 100 μm. n = 3. $^{\#}P < 0.05$, compared to the CON group, $^{*}P < 0.05$, compared to the 0 μmol/L BPA group. Arg-1: arginase-1; BPA:

Bisphenol A; iNOS: inducible nitric oxide synthase; CD86: cluster of differentiation 86; IL-6: interleukin-6; TNF-α: tumor necrosis factor-α; mRNA: messenger ribonucleicacid; DAPI: 4':6-diamidino-2-phenylindol; CON: control; LPS: lipopolysaccharide.

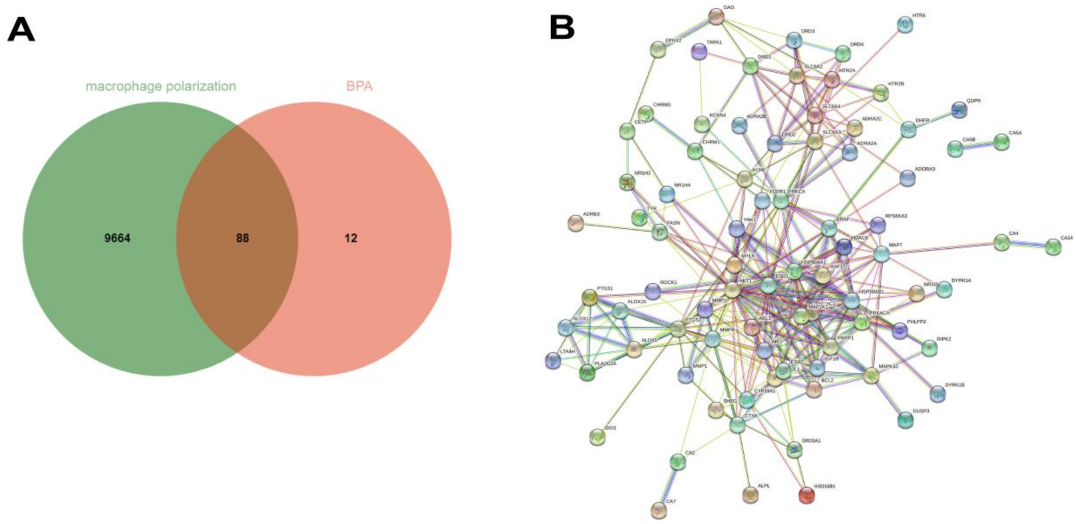

Supplementary Figure S2: Analysis of the common targets of BPA and macrophage polarization. (A) 88 common targets were screened out between BPA and macrophage polarization using online websites. (B) A PPI network map of BPA and polarization in macrophages was constructed. BPA: Bisphenol A; PPI: protein-protein interaction.

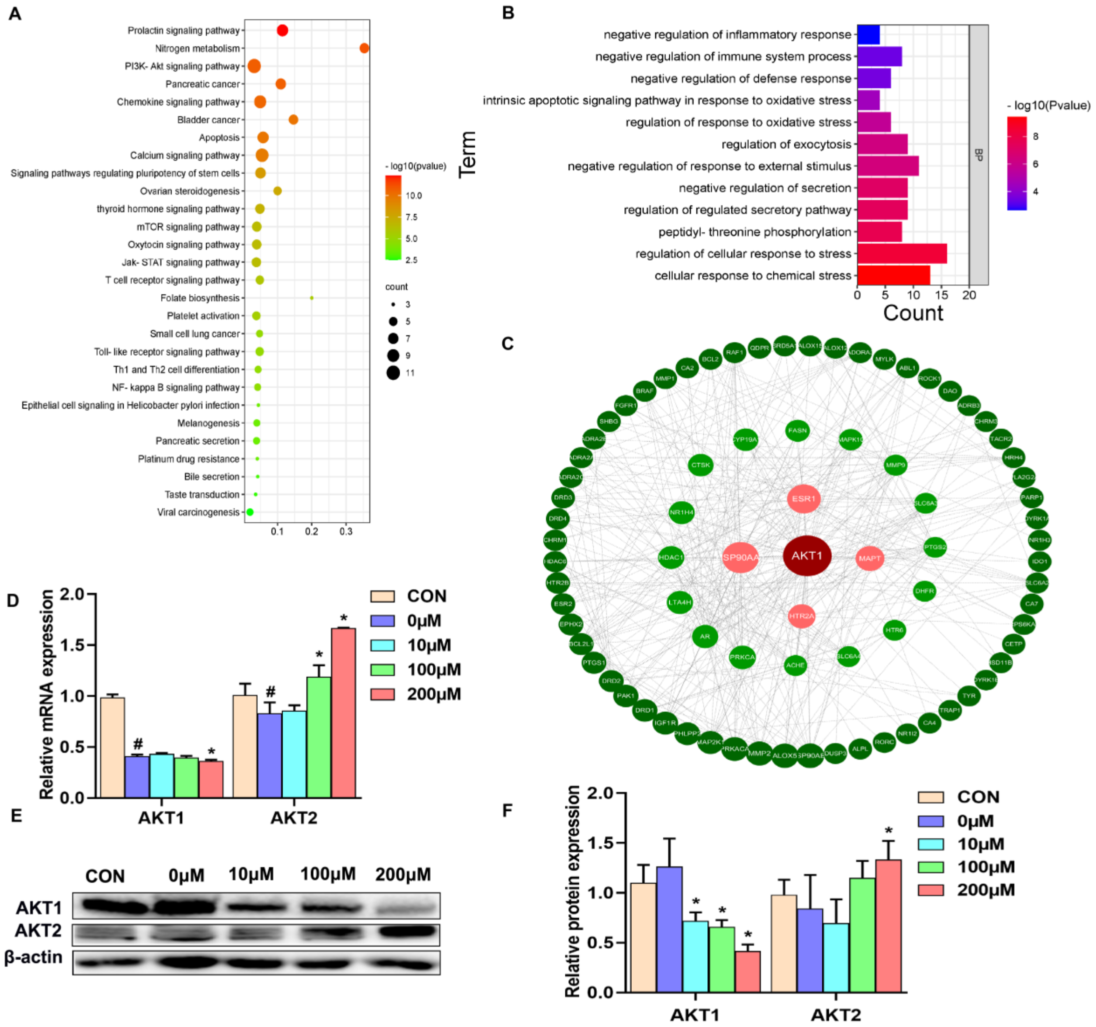

Supplementary Figure S3: BPA affects the expression of 88 macrophage polarization-related genes through bioinformatics analysis. (A) KEGG analysis of the 88 co-expressed targets. (B) GO analysis of the biological processes involved in the 88 common targets. (C) The protein-protein interaction analysis of the 77 co-expressed targets visualized by Cytoscape. (D-F) RAW264.7 cells were treated with BPA (0, 10, 100, and 200 μmol/L) with 10 μg/mL LPS for 12 h. (D) The relative mRNA levels of AKT1 and AKT2 were detected by qRT-PCR. (E, F) The protein levels of AKT1 and AKT2 were tested by Western blot. $n = 3$. $^{\#}P < 0.05$, compared to the CON group, $^{*}P < 0.05$, compared to the 0 μmol/L BPA group. BPA: Bisphenol A; GO: Gene Ontology; KEGG: Kyoto Encyclopedia of Genes and Genomes; mRNA: messenger ribonucleicacid; AKT: Protein kinase B; mTOR: mechanistic target of rapamycin; LPS: lipopolysaccharide; qRT-PCR: quantitative reverse transcription polymerase chain reaction; CON: control.

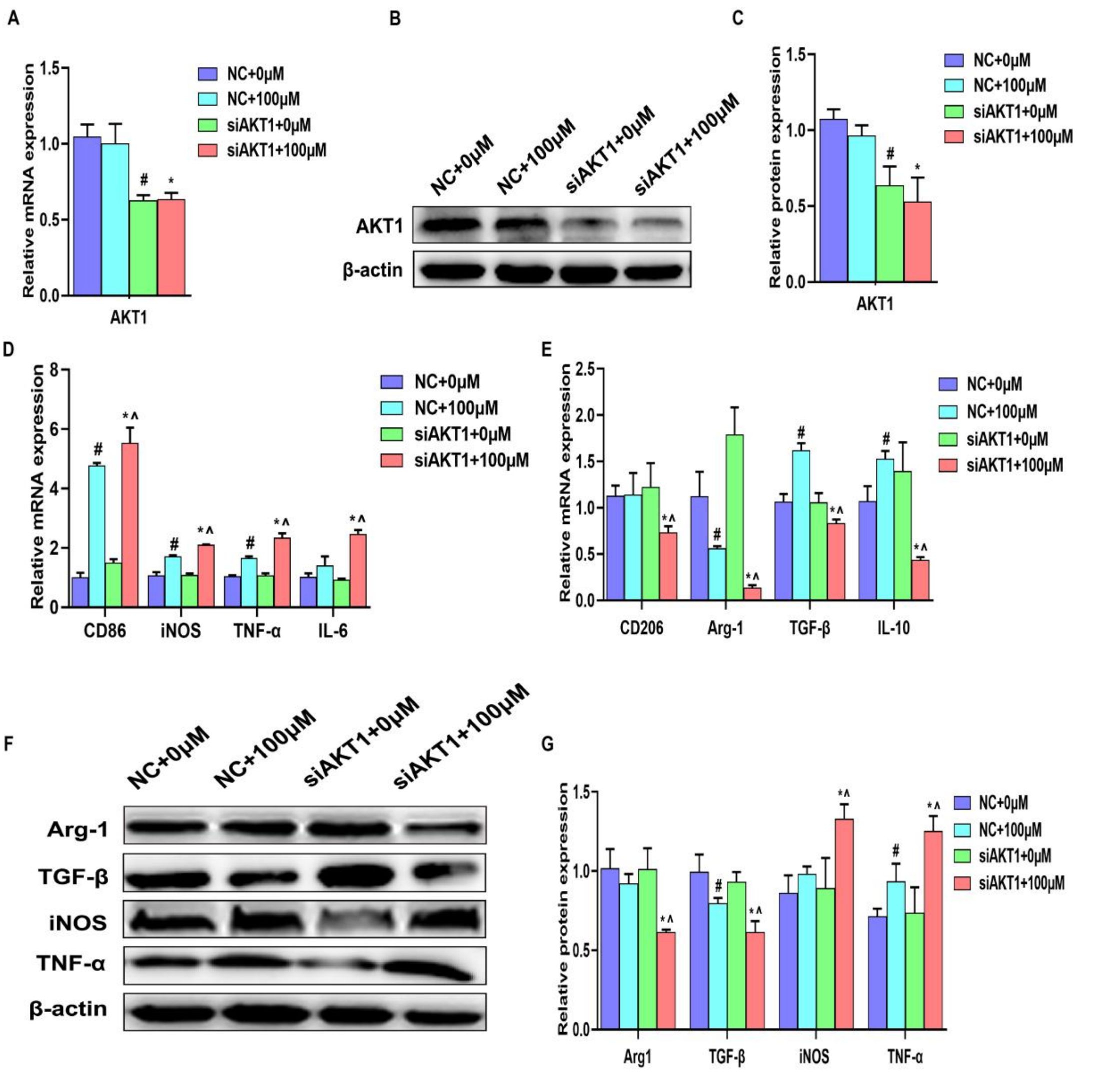

Supplementary Figure S4: Knockdown of AKT1 promotes the BPA-induced M1-type polarization and inhibits the M2-type polarization in macrophages. RAW264.7 cells were transfected with siAKT1 or NC siRNA for 24 h or 48 h, then treated with BPA (0, 100 μmol/L) and 10 μg/mL LPS for 12 h. (A) The mRNA level of AKT1. (B, C) Western blot analysis of the protein level of AKT1. (D) The mRNA levels of CD86, iNOS, and pro-inflammatory cytokines were analyzed by qRT-PCR. (E) The mRNA expression of CD206, Arg-1, and anti-inflammatory cytokines. (F, G) The protein levels of Arg-1, TGF-β, iNOS, and TNF-α. $n = 3$. $^{\#}P < 0.05$, compared to the NC + 0 μmol/L BPA group, $^{*}P < 0.05$, compared to the NC + 100 μmol/L BPA group, $^{\wedge}P < 0.05$, compared to the siAKT1 + 0 μmol/L BPA group. Arg-1: arginase-1; BPA: Bisphenol A; mRNA: messenger ribonucleicacid; AKT: protein kinase B; qRT-PCR: quantitative reverse transcription polymerase chain reaction; CON: control; siAKT1: small interfering protein kinase B 1; iNOS: inducible nitric oxide synthase; CD86: cluster of differentiation 86; TNF-α: tumor necrosis factor-α; TGF-β: transforming growth

factor-β; siRNA: small interfering ribonucleicacid; NC: negative control; LPS: lipopolysaccharide.

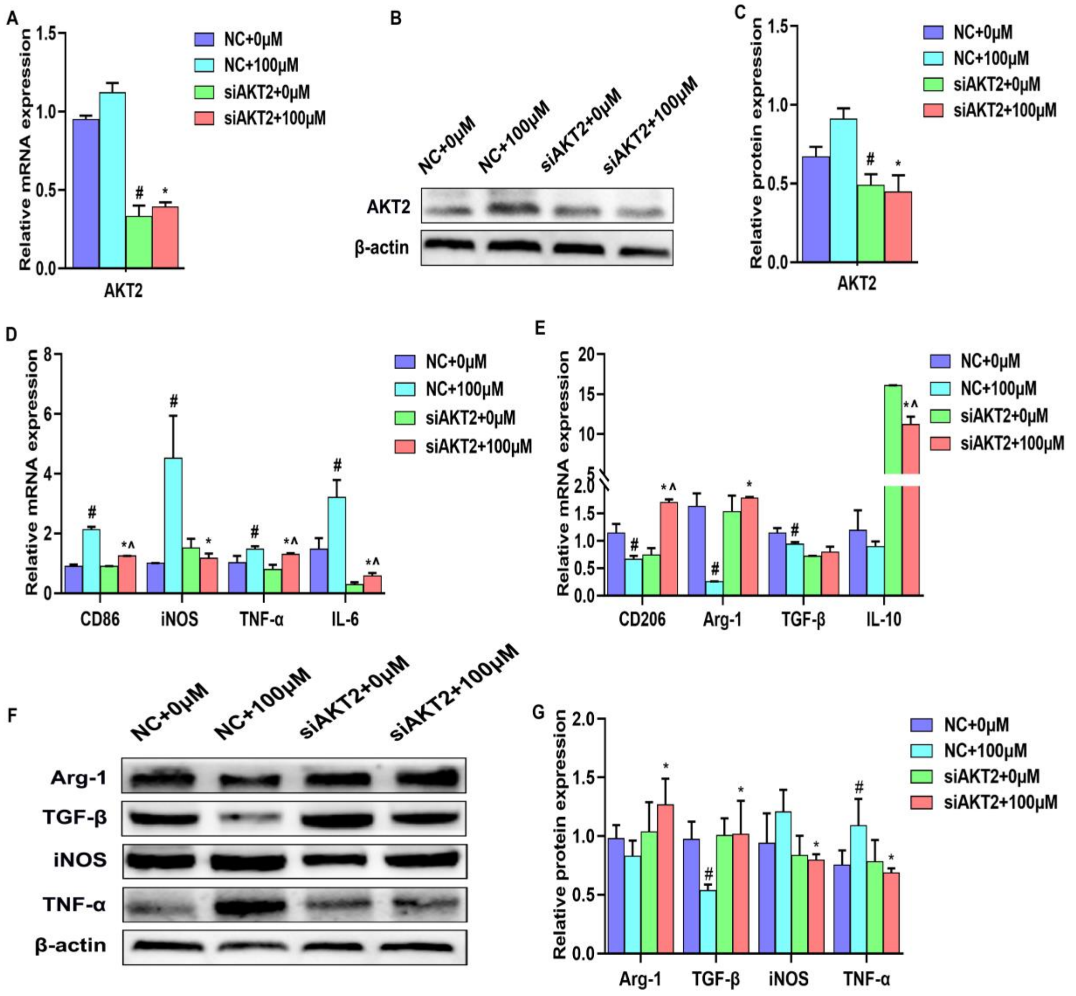

Supplementary Figure S5: Knockdown of AKT2 suppresses M1 polarization and enhances M2 polarization in macrophages. RAW264.7 cells were transfected with siAKT2 or NC siRNA for 24 h or 48 h, then treated with BPA (0, 100 μmol/L) and 10 μg/mL LPS for 12 h. (A) The expression of AKT2 mRNA. (B, C) The expression of AKT2 protein. (D, E) The mRNA levels of CD86, iNOS, pro-inflammatory cytokines, CD206, Arg-1, and anti-inflammatory cytokines. (F, G) The protein levels of Arg-1, TGF-β, iNOS, and TNF-α. $n = 3$. $^{\#}P < 0.05$, compared to the NC + 0 μmol/L BPA group, $^{*}P < 0.05$, compared to the NC + 100 μmol/L BPA group, $^{\wedge}P < 0.05$, compared to the siAKT2 + 0 μmol/L BPA group. Arg-1: arginase-1; BPA: Bisphenol A; GO: mRNA: messenger ribonucleicacid; AKT: protein kinase B; qRT-PCR: quantitative reverse transcription polymerase chain reaction; CON: control; siAKT2: small interfering protein kinase B 2; iNOS: inducible nitric oxide synthase; CD86: cluster of differentiation 86; TNF-α: tumor necrosis factor-α; TGF-β: transforming growth

factor-β; siRNA: small interfering ribonucleicacid; NC: negative control; LPS: lipopolysaccharide.

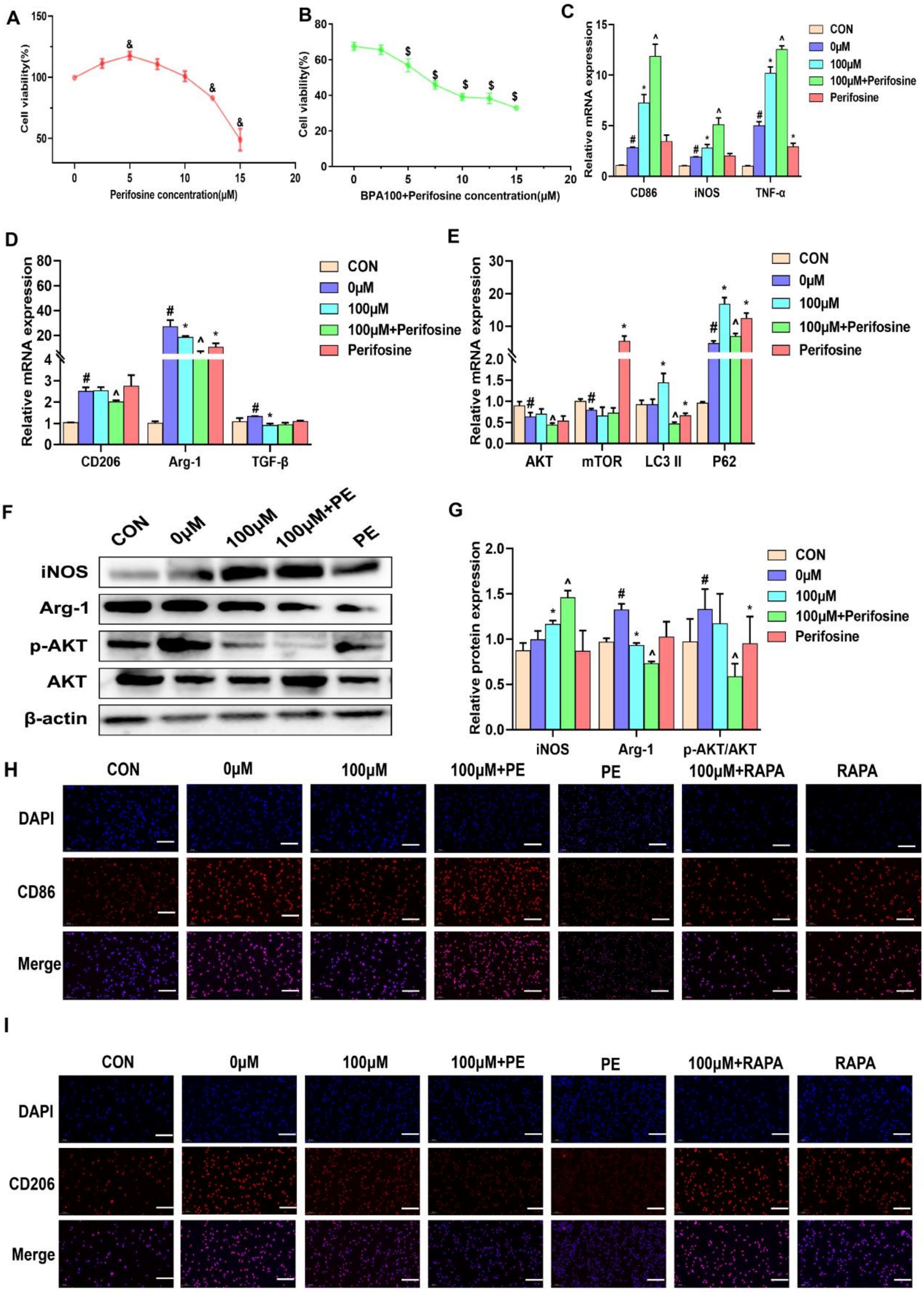

Supplementary Figure S6: PE increases M1 macrophage polarization and decreases M2 macrophage polarization via mTOR-dependent autophagy. (A, B) RAW264. 7 cells

were treated with a range of PE concentrations (0, 2.5, 5, 7.5, 10, 12.5, and 15 μmol/L), 100 μmol/L BPA and 10 μg/mL LPS for 12 h. (A) Effect of PE on cell viability. (B) Cell viability by the combination of 100 μmol/L BPA and PE. (C-I) RAW264.7 cells were treated with 10 μg/mL LPS and 100 μmol/L BPA or 5 μmol/L PE for 12 h. (C) M1-type-related genes, (D) M2-type-related genes, (E) AKT, mTOR and autophagy-related genes expression detected by qRT-PCR. (F, G) Western blot analysis of iNOS, Arg-1, AKT and p-AKT. (H, I) The protein levels of CD86 and CD206 were detected by immunofluorescence staining. Scale bar represents 100 μm. n = 3. $^{\&}P < 0.05$, compared to the 0 μmol/L perifosine group, $^{\$}P < 0.05$, compared to the BPA 100 μmol/L + 0 μmol/L perifosine group, $^{\#}P < 0.05$, compared to the CON group, $^{*}P <$ 0.05, compared to the 0 μmol/L BPA group, $^{\wedge}P < 0.05$, compared to the 100 μmol/L BPA group. BPA: Bisphenol A; mTOR: mechanistic target of rapamycin; PE: perifosine; iNOS: inducible nitric oxide synthase; CD86: cluster of differentiation 86; AKT: protein kinase B; Arg-1: arginase-1; CON: control; DAPI: 4':6-diamidino-2-phenylindol; qRT-PCR: quantitative reverse transcription polymerase chain reaction; mRNA: messenger ribonucleicacid; p-AKT: phosphorylated protein kinase B; RAPA: rapamycin; LPS: lipopolysaccharide.

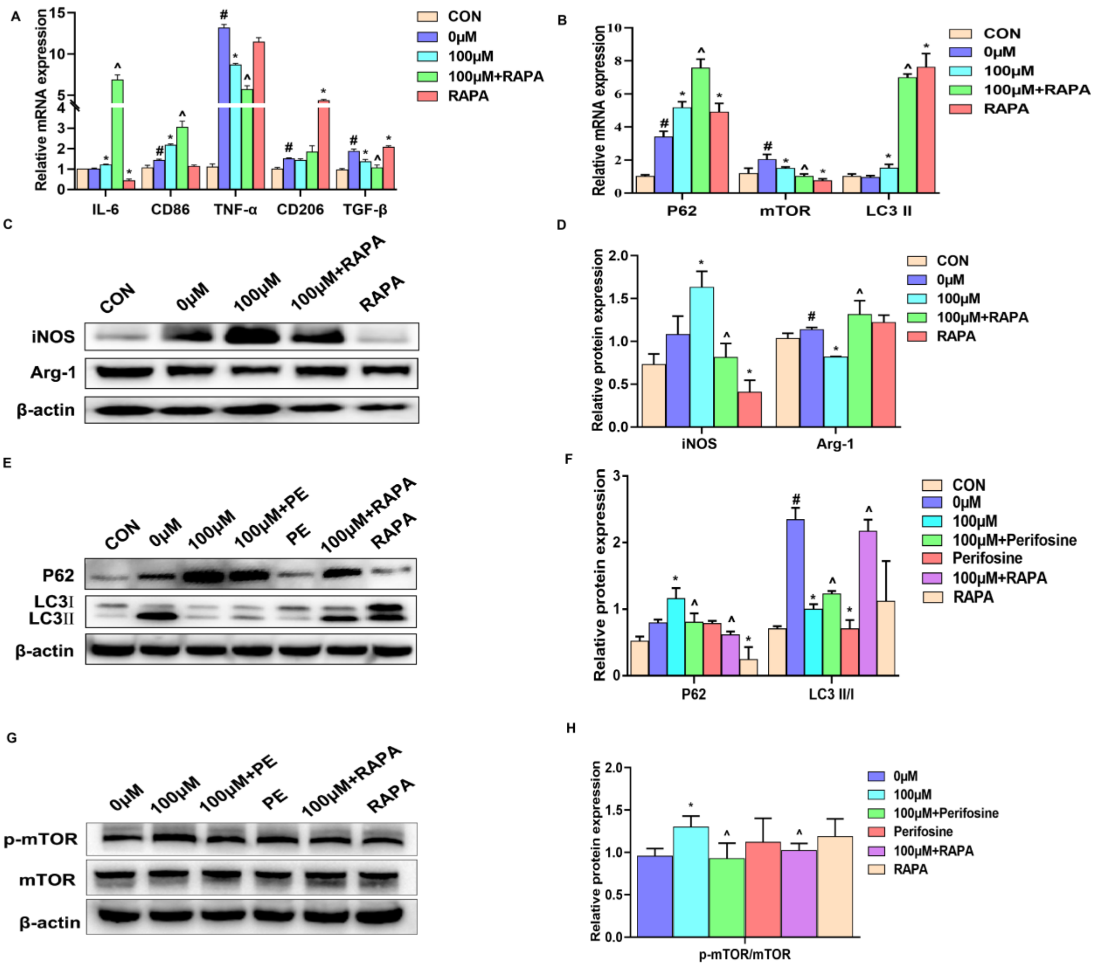

Supplementary Figure S7: Effect of RAPA on macrophage polarization through activating autophagy. (A, B) RAW264.7 cells were treated with 10 μg/mL LPS and 100 μmol/L BPA or RAPA for 4 h. (A) Inflammatory cytokines and (B) autophagy-related genes expression determined by qRT-PCR. (C-F) RAW264.7 cells were treated with 10 μg/mL LPS and 100 μmol/L BPA or RAPA for 12 h. (C, D) iNOS and Arg-1 protein expression analyzed by Western blot. (E-H) The protein expression levels of P62, LC3II/I, p-mTOR, and mTOR. $n = 3$. $^{\#}P < 0.05$, compared to the CON group, $^{*}P < 0.05$, compared to the 0 μmol/L BPA group, $^{\wedge}P < 0.05$, compared to the 100 μmol/L BPA group. mRNA: messenger ribonucleicacid; CD86: cluster of differentiation 86; TNF-α: tumor necrosis factor-α; TGF-β: transforming growth factor-β; CON: control; RAPA: rapamycin; iNOS: inducible nitric oxide synthase; Arg-1: arginase-1; PE: perifosine; mTOR: mechanistic target of rapamycin; p-mTOR: phosphorylated mammalian target of rapamycin; LC3I: microtubule-associated proteins 1A/1B light chain 3 I; P62: Sequestosome 1; BPA: Bisphenol A; qRT-PCR: quantitative reverse transcription polymerase chain reaction; LPS: lipopolysaccharide.
